# Association between individual-level and community-level socio-economic status and blood pressure among Inuit in Greenland

**DOI:** 10.3402/ijch.v75.32757

**Published:** 2016-12-08

**Authors:** Mylène Riva, Christina Viskum Lytken Larsen, Peter Bjerregaard

**Affiliations:** 1 Institute for Health and Social Policy and Department of Geography, McGill University, Montreal, Canada; 2 National Institute of Public Health, University of Southern Denmark, Copenhagen, Denmark; 3 Greenland Centre for Health Research, University of Greenland, Nuuk, Greenland

**Keywords:** blood pressure, indigenous populations, Inuit, residence characteristics, small-area analysis, Greenland

## Abstract

**Background:**

Despite abundant evidence that socio-economic status (SES) is a fundamental determinant of health, there is a dearth of research examining association between SES, measured at the individual and community levels, and cardiovascular risk factors and morbidity among indigenous populations.

**Objectives:**

To examine the influence of individual-level and community-level SES on systolic and diastolic blood pressure among Greenlandic Inuit.

**Methods:**

Multilevel analysis of cross-sectional data from the Inuit Health in Transition – Greenland Survey, to which 3,108 Greenlandic Inuit aged 18 years and older participated. Blood pressure is measured using an automatic device, according to standardized protocol. Individual SES is measured by education. Community socio-economic conditions are measured using combined information on average disposable household income and settlement type.

**Results:**

Education was not significantly associated with blood pressure. There was an inverse U-shape association between community socio-economic conditions and blood pressure with significantly lower SBP and DBP among participants living in remote traditional villages characterized by lower average disposable household income and in affluent more urbanized towns. Sex-stratified analyses demonstrate the salience of community conditions for men.

**Conclusions:**

The association observed between blood pressure and community-level socio-economic conditions suggests that public health and social policies, programmes and interventions aiming to improve living conditions might improve cardiovascular health in Greenland. Studies are required to further examine social gradients in cardiovascular risk factors and morbidity among indigenous populations using different measures of SES.

Despite abundant evidence that socio-economic status (SES) is a fundamental determinant of health, there is a dearth of research examining association between SES, measured at the individual and community levels and cardiovascular risk factors and morbidity (and health more largely) among indigenous populations (1,2). A recent review of studies examining social gradients in relation to various health measurements (physical and mental health outcomes; objective and self-reported health) among Indigenous Australians concluded that “in contrast to the ubiquitous, strong associations between SES and health in the general population, there is a less universal and less consistent SES patterning in Indigenous Australian health” (p. 2012) (2).

Higher education, income, employment and housing statuses, often used as individual measures of SES, are often shown to be associated with better health outcomes in non-indigenous populations. In indigenous populations, studies examining socio-economic inequalities in health outcomes have reported mixed results. Shepherd et al. discussed the possibility of different associations between “traditional” SES indicators and health outcomes among indigenous populations because of persistent issues of exclusion, marginalization and discrimination leading to less pronounced social gradient in health (2). Other factors may bear greater importance for health of indigenous peoples’, for example, cultural continuity (3) and connection to traditional land (4,5) and social support (6,7).

In Greenland, higher degrees of social transition (measured using childhood and current place of residence, employment type and education) was associated with increased prevalence of clinical and biochemical risk factors for cardiovascular diseases (CVD) (8). Among Inuit in Nunavut, Canada, the risk of overweight and obesity was greater among individuals with higher socio-economic position (e.g. higher education, in employment, higher income and living in private housing) (9). Other studies have reported an inverse socio-economic gradient, with higher CVD risks among people in less favourable socio-economic circumstances. Among a First Nation in Ontario, Canada, CVD rates and the burden of risk factors (combined information on hypertension, diabetes, smoking, hyperlipidaemia and obesity) were higher among those with lower income (10). In indigenous populations in Australia, higher cardiovascular morbidity was associated with being unemployed (11), whereas elevated risk of diabetes was observed among those reporting lower income, not working and renting (vs. owning) their house (12).

To better understand the SES–health relationship in indigenous context, Shepherd et al. pointed to the importance of multilevel studies to document SES simultaneously across different levels, that is, individual, household and community. This is in accordance with indigenous worldviews, which recognize the intertwining of peoples’ health to that of their family and environment (13). Frameworks have been proposed to conceptualize and operationalize the influence of community conditions on cardiometabolic diseases among indigenous populations (1,14), but few studies to date have investigated the multilevel influence of socio-economic conditions on health among indigenous peoples.

Among non-indigenous and mostly urban populations, several systematic literature reviews have documented associations between community socio-economic deprivation, built environment features, and urban–rural location and CVD (15,16) and risk factors such as obesity (17–20), diabetes and blood pressure/hypertension (21). These associations persist after controlling for individual-level characteristics such as SES and behavioural risk factors, suggesting that local community conditions play a role in shaping the distribution of health. Among indigenous populations, the few studies examining community conditions in relation to cardiovascular health and risk factors have produced mixed results. Area-level socioeconomic conditions have been associated with elevated standardized prevalence of diabetes among First Nations in the province of Manitoba, Canada (22), and with elevated standardized prevalence of CVD among Māori in New Zealand (23). Another study reported a U-shaped association between area-level deprivation and standardized prevalence of diabetes among Māori, with greater prevalence of diabetes observed in both lower and higher quintiles of area deprivation (24). With regards to geographic location, higher rates of diabetes were observed in more remote communities in Greenland (25), whereas a study conducted among Indigenous Australians reported a complex patterning of cardiometabolic risk and disease between urban, peri-urban and remote communities (11). A recent multilevel study conducted in Australia showed higher rates of acute myocardial infarction (AMI) among Indigenous peoples living in more disadvantaged and remote areas, and wider disparities in AMI rates between Indigenous and non-Indigenous Australians with remoteness and area-level socioeconomic disadvantage (26); this study, however, did not account for individual-level SES.

Social (between people) and geographic (between communities/regions) inequalities in cardiovascular risk factors and morbidity highlight the heterogeneity in living conditions experienced by indigenous populations worldwide. Still, relatively little is known about the multilevel and joint influence of individual-level and community-level SES on cardiovascular health and risk factors (27). For public health surveillance and health promotion, documenting and understanding the distribution of cardiovascular risks and diseases (and health more holistically defined) between people and between places bear critical implications in increasing the knowledge about the aetiological significance of contextual dimensions for health. It also contributes knowledge to support the formulation and implementation of interventions designed to reach large groups of people (even entire populations) by changing the conditions of living environments to make them more conducive to health.

Thought to be traditionally protected from CVD because of particular genetic endowment, high dietary intake of marine mammals and fish, and vigorous physical activity (28), Inuit populations are increasingly burdened by CVD. This situation is attributed to rapid social, nutrition, cultural and environmental transitions experienced by Inuit populations and characterized by the rising prevalence of behavioural risk factors (smoking, low physical activity and westernized diet), obesity and hypertension (28–30). Using data from a large cross-sectional health survey conducted in Greenland, this study has three objectives: (a) to assess the extent of variation in blood pressure across communities in Greenland; (b) to examine the association between individual SES and community-level socio-economic conditions with blood pressure; and (c) to examine whether these associations are similar for men and women.

## Methods

### Setting of the study

Greenland was under Danish colonial system between 1721 and 1953. It attained Home Rule in 1979 and has a self-governing status since 2009. Urbanization started in the early 20th century and increased rapidly since the 1950s. In 1951, 68% of the population lived in villages with less than 500 inhabitants; by 2010, this proportion decreased to 15%. Today, the total population of Greenland is about 56,400 of whom 90% are Greenlanders (Inuit) living in one of 80 communities located on a narrow coastal strip (31,32). There are no roads connecting the communities. A town is defined historically as the largest community in each of the 17 districts. In 2010, the population of the towns varied between 469 and 5,460, with 15,469 residents in Nuuk, the capital. Despite its small size, Nuuk has resources similar to those of northern capitals, such as central government offices, post-secondary teaching institutions including a university, and the central hospital for Greenland. District school(s), health centre or hospital, church, district administration and main shops are located in the towns; these institutions are absent or present to a much smaller extent in villages. Population in villages varies from less than 10 to around 550. The most remote communities in Greenland are located on the East Coast and in the far North; they are characterized by extreme remoteness, dialects considerably different from Central West Greenlandic, lower income and employment opportunities.

### Study design and data collection

Data for this cross-sectional multilevel study are from the *Inuit Health in Transition – Greenland Survey*. A full description of the study methods is available elsewhere (33); they are only summarized here. Data were collected as part of a countrywide cross-sectional health survey in Greenland over 2005–2010. Adult participants aged 18 years and older and born in Greenland or Denmark were selected as a stratified random sample. Greenland was divided into strata based on region (South West coast; Central West coast; North West coast; East Greenland; North Greenland) and community type (larger towns with ≥2,000 inhabitants; smaller towns with <2,000 inhabitants; villages with <500 inhabitants). From each of these strata, one or more towns, and two or three villages, were selected for the study as being representative of the stratum with regard to living conditions. A random sample was drawn from the central population register to obtain around 300 participants from each town; this number represents the practical limit for a research team during a 4- to 6-week visit. Villages were chosen at random in the strata, and in the selected villages, all adults were invited to participate. More than one person per household could participate in the survey.

Data were collected using clinical procedures, sampling of biological media and interviewer- and self-administered questionnaires collecting information on socio-demographic factors, general physical and mental health problems and behaviours. Questionnaires were developed in Danish, translated into Greenlandic, back translated and revised. Interviews were conducted in the language of choice of the participant, most often in Greenlandic, by native Greenlandic speaking interviewers who had been trained in the study procedures. Ethnicity as Greenlander or Dane was determined at enrolment based on self-identification and primary language. A total of 3,108 Greenlanders living in 9 towns and 13 villages participated to the survey with a participation rate of 66.7%. The study surveyed 9.2% of the adult, Greenland born population. Ethical approval was received from the Committee for Research Ethics in Greenland. Participants gave their written consent after being informed about the study orally and in writing.

### Measures

Systolic blood pressure (SBP) and diastolic blood pressure (DBP) were measured on the right arm of the sitting participant after at least 5 min of initial rest, using an automatic measuring device (Kivex UA-779) with appropriate cuff size. SBP and DBP were read to the nearest mm Hg three times, with at least 1-min interval between measurements. The last two measurements were averaged for the analyses.

Individual SES was measured using information on education. Participants reported their highest level of schooling, which was grouped in three categories: high school or less, vocational training after high school and higher education (which corresponds to university or professional training, e.g. nursing and teaching).

Community socio-economic conditions were measured using information on average disposable household income for 2008 “equivalized” to account for the total number of person per household (2008 was selected as it is the mid-point in the survey data collection). This information was retrieved from Statistics Greenland and appended to the survey file based on community identifier (www.stat.gl/default.asp?Lang=en). The 22 communities were categorized in tertiles of equivalized average disposable household income (i.e. low, average and high SES). Communities were further categorized by settlement type, where towns, including larger and smaller towns, were contrasted to villages. As settlement type and community socio-economic conditions are strongly correlated (χ^2^=2,100, p<0.001; results not tabulated), we created a composite measure categorizing towns and villages by their socio-economic conditions. Communities are thus categorized in one of four types: town with average SES (reference category in the analysis), town with high SES, village with low SES or village with average or high SES. There were no towns within lower tertiles of average disposable income.

Individuals’ characteristics considered included sex, age (modelled as a quadratic variable to account for non-linear association with blood pressure), fluency in speaking Danish (used as a proxy for cultural change) (34), treatment with antihypertensive drugs (self-reported in the individual questionnaire and assessed by nurses in the clinical assessment) and self-reported family history of hypertension (in parents or siblings). We did not adjust for other risk factors such as smoking or body mass index, as these may be on the pathway between SES and blood pressure (35).

### Statistical analyses

Data were analysed using multilevel linear models. This type of analysis allows partitioning the variation in outcome variables, here SBP and DBP measured at the individual level, into two (or more) sources: variation attributable to differences between individuals and variation attributable to differences between communities. In doing so, multilevel models account for the clustering of similar individuals within communities (non-independence of observations within groups) and allow for the assessment of the relative contribution of both individual-level and community-level characteristics to health variation (36). For this study, participants were clustered in two groups: households (for 25% of the sample, more than one person per household participated to the survey) and communities. Variation in participants’ blood pressure between communities was assessed using unconditional (unadjusted) model. Association between blood pressure and individual-level SES was first modelled, adjusting for other individuals’ characteristics (model 1). Then, community socio-economic conditions were entered into the model (model 2). A p-value of <0.05 is used to determine statistical significance. Analyses were performed using Stata 11.0 (37).

## Results

Descriptive statistics of the study sample appear in Table I. More women (56%) than men participated, and 55% of participants were aged 45 years and older. Less than 25% of the sample lived in villages and 17% in communities categorized as most deprived. In 2008, overall mean equivalized household income was 119,125 DKK (16,336 USD in 2008); it was 143,457 DKK ($18,791 USD in 2008) in towns and 102,280 DKK ($13,400 USD in 2008) in villages (t=4.28; p<0.001; results not tabulated). Overall, mean SBP was 130 mmHg, with 26% of participants having an SBP ≥140 mmHg. Mean DBP was 79 mmHg, with 18% of participants having a DBP ≥90 mmHg. Thirty-eight percent of participants were classified as having hypertension (defined as blood pressure ≥140/90 mm Hg or treatment with antihypertensive drugs) (38).

**Table I:** Descriptive statistics of the participants, Inuit Health in Transition – Greenland Survey 2005–2010 (*n*=3,108)

Blood pressure		
Systolic blood pressure in mmHg, mean (SD)		129.88 (20.13)
Diastolic blood pressure in mmHg, mean (SD)		78.93 (12.80)
Hypertension, % (n)		0.38 (1,171)
Individual characteristics		
Sex (women), % (n)		0.56 (1,737)
Age, years, mean (SD)		44.43 (14.83)
Education High school education or less		0.60 (1,851)
Vocational training		0.29 (897)
Higher education		0.10 (307)
Language – Speaks Danish with difficulty or not at all, % (n)		0.43 (1,345)
Use of antihypertensive medication, % (n)		0.14 (434)
Family history of hypertension, % (n)		0.23 (716)
Tertiles of community-level equivalized average household income (SES)		
by settlement type, % (n)^a^	Towns; 0.76 (2,368)	Villages; 0.24 (740)
Lower SES (Mean DKK [95% CI]: 90,662 [80,813–100,509])^b^	0 (0)	0.71 (525)
Middle SES (Mean DKK [95% CI]: 119,475 [113,162–125,788])^c^	0.30 (713)	0.20 (150)
Higher SES (Mean DKK [95% CI]: 151,306 [132,377–170,234])^d^	0.70 (1,655)	0.09 (65)

a Sample size of participants within community categorization;

b Mean (95% CI), in 2008 USD: $12,434 ($11,083–$13,784);

c Mean (95% CI), in 2008 USD: $16,385 ($15,519–$17,251);

d Mean (95% CI), in 2008 USD: $20,751 ($18,155–$23,346).

**Fig. 1:**
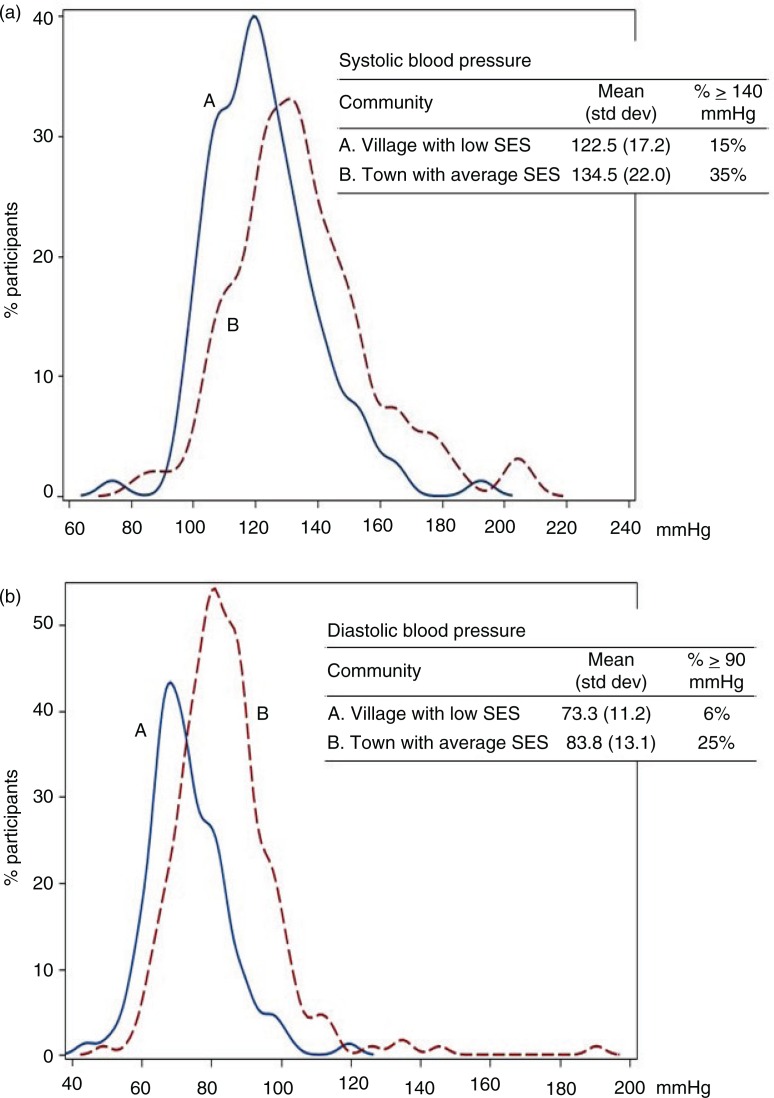
Unadjusted variation in the distribution of (a) SBP and (b) DBP among participants living in two communities categorized as A. a village with low SES and B. a town with average SES. Inuit Health in Transition – Greenland Survey 2005–2010.

There was a significant variation in participants’ blood pressure between communities in Greenland, as indicated by results of the unadjusted multilevel models. About 2.2% of the total variation in SBP (χ^2^=26.96; p<0.001) and 5.2% of total variation in DBP (χ^2^=105.65; p<0.001) were attributed to differences between communities (results not tabulated). To illustrate this variation, Fig. 1 plots the unadjusted variation in the distribution of SBP and DBP among participants living in two of the 22 communities involved in the survey; community A corresponds to a village with low SES and community B to a town with average SES (reference category for regression analysis). The means of the distribution of SBP and DBP are both lower in community A compared to community B. For example, in the village with low SES, mean SBP is 122.5 mmHg and 15% of participants present an SBP above 140 mmHg. In contrast, mean SBP is 134.8 mmHg and 35% of participants have an SBP above 140 mmHg in the town with average SES. This variation in blood pressure between communities could be attributed to individuals’ characteristics and/or to community conditions.

Results of association between blood pressure and individuals’ SES as measured by education (model 1) are presented in Table II; these models are adjusted for participants’ characteristics. Education was not significantly associated with blood pressure at conventional p<0.05. Adjusting for other covariates did not fully explain the between-community variation in SBP and DBP, which remained statistically significant.

**Table II:** Multilevel associations (coefficient [coeff] and 95% confidence intervals [95% CI]) between systolic and diastolic blood pressure and individual-level and community-level socio-economic status (SES), adjusting for individual characteristics, Inuit Health in Transition – Greenland Survey 2005–2010; *n*=2,992

	Systolic blood pressure		Diastolic blood pressure	
		
	Model 1, Coeff (95% CI)	Model 2, Coeff (95% CI)	Model 1, Coeff (95% CI)	Model 2, Coeff (95% CI)
Education				
High school or less	1.00	1.00	1.00	1.00
Vocational training	0.98 (−0.60, 2.56)	1.02 (−0.55, 2.59)	0.91 (−0.15, 1.98)†	0.91 (−0.15, 1.98)†
Higher education	−0.38 (−2.73, 1.97)	−0.11 (−2.45, 2.23)	−0.35 (−1.93, 1.23)	−0.31 (−1.90, 1.27)
Community characterization				
Town, mid SES		1.00		1.00
Town, high SES		−3.61 (−5.24, −1.98)***		−4.11 (−6.71, −1.51)**
Village, low SES		−3.34 (−5.45, −1.22)**		−4.91 (−7.62, −2.19)***
Village, middle/high SES		−1.69 (−4.48, 1.11)		−3.97 (−7.09, −0.85)*
Sex				
Men	1.00	1.00	1.00	1.00
Women	−8.77 (−10.06, −7.48)***	−8.77 (−10.06, −7.48)***	−3.59 (−4.46, −2.72)***	−3.59 (−4.46, −2.73)***
Age	−0.39 (−0.63, −0.15)**	−0.41 (−0.65, −0.17)**	0.91 (0.75, 1.07)***	0.91 (0.75, 1.07)***
Age squared	0.01 (0.01, 0.01)***	0.01 (0.01, 0.01)***	−0.01 (−0.01, −0.01)***	−0.01 (−0.01, −0.01)***
Speaks Danish				
Yes	1.00	1.00	1.00	1.00
No	−1.49 (−2.98, 0.01)†	−1.49 (−3.00, 0.02)†	−1.80 (−2.82, −0.79)**	−1.74 (−2.77, −0.72)**
Use of antihypertensive medication				
No	1.00	1.00	1.00	1.00
Yes	7.26 (5.23, 9.28)***	7.29 (5.27, 9.31)***	3.15 (1.79, 4.52)***	3.15 (1.79, 4.52)***
Family history of hypertension				
No	1.00	1.00	1.00	1.00
Yes	2.77 (1.23, 4.32)***	2.66 (1.13, 4.20)**	1.49 (0.45, 2.54)**	1.49 (0.45, 2.53)**
Variance component	χ^2^=11.48; p=0.003	χ^2^=0.45; p=0.797	χ^2^=82.51; p<0.001	χ^2^=14.16; p=0.001

† *p*<0.10;

* *p*<0.05;

** *p*<0.01;

*** *p*<0.001.

There was a direct and statistically significant association between community socio-economic conditions and blood pressure (model 2; Table II). Compared to people living in towns characterized by average socio-economic conditions, SBP was 3.61 mmHg lower (p<0.001) in affluent towns and 3.34 mmHg lower (p=0.002) in villages characterized by low SES. DBP was significantly lower among participants living in affluent towns and in villages either characterized by low SES or by average or high SES. Accounting for community socio-economic conditions explained the between-community variation in SBP, which was no longer significant. Significant variation between communities remained for DBP (p=0.001).

In sex-stratified analysis (Table III), there was a tendency for community socio-economic conditions to be more strongly associated with blood pressure among men. It is to note, however, that confidence intervals in the men-specific models are wide, requiring caution when interpreting these results.

**Table III:** Sex-stratified multilevel associations (coefficient [coeff] and 95% confidence intervals [95% CI]) between systolic and diastolic blood pressure and individual-level and community-level socio-economic status (SES), adjusting for individual characteristics, Inuit Health in Transition – Greenland Survey 2005–2010; *n*=1,294 for men, and *n*=1,648 for women

	Systolic blood pressure		Diastolic blood pressure	
		
	Men, Coeff (95% CI)	Women, Coeff (95% CI)	Men, Coeff (95% CI)	Women, Coeff (95%CI)
Education				
High school or less	1.00	1.00	1.00	1.00
Vocational training	1.91 (−0.47, 4.28)	0.98 (−1.13, 3.10)	1.49 (−0.16, 3.13)†	0.14 (−1.26, 1.54)
Higher education	−0.05 (−3.72, 3.61)	0.59 (−2.43, 3.61)	−0.45 (−2.98, 2.08)	−0.27 (−2.27, 1.73)
Community characterization				
Town, mid SES	1.00	1.00	1.00	1.00
Town, high SES	−5.49 (−9.19, −1.80)**	−2.28 (−4.65, 0.09)†	−4.69 (−8.28, −1.10)*	−3.89 (−5.64, −2.14)***
Village, low SES	−6.08 (−10.27, −1.89)**	−1.19 (−4.12, 1.74)	−6.07 (−9.89, −2.26)**	−4.53 (−6.63, −2.44)***
Village, middle/high SES	−4.80 (−9.82, 0.22)†	1.06 (−2.79, 4.92)	−5.40 (−9.77, −1.02)*	−3.16 (−5.84, −0.47)*
Age	−0.18 (−0.58, 0.21)	−0.54 (−0.84, −0.25)***	1.29 (1.02, 1.56)***	0.66 (0.47, 0.86)***
Age squared	0.01 (0.00, 0.01)*	0.01 (0.01, 0.02)***	−0.01 (−0.02, −0.01)***	−0.01 (−0.01, 0.00)***
Speaks Danish				
Yes	1.00	1.00	1.00	1.00
No	−1.46 (−3.83, 0.91)	−1.15 (−3.10, 0.79)	−0.67 (−2.31, 0.96)	−2.40 (−3.69, −1.11)***
Use of antihypertensive medication				
No	1.00	1.00	1.00	1.00
Yes	7.69 (4.23, 11.16)***	6.86 (4.42, 9.30)***	4.84 (2.45, 7.23)***	2.22 (0.60, 3.83)**
Family history of hypertension				
No	1.00	1.00	1.00	1.00
Yes	2.58 (0.01, 5.16)*	2.86 (0.97, 4.75)**	0.94 (−0.84, 2.72)	1.82 (0.57, 3.07)**

† *p*<0.10;

* *p*<0.05;

** *p*<0.01;

*** *p*<0.001.

## Discussion

While social determinants are recognized as key factors leading to health (or ill-health) in the scientific literature and public policy, less attention has been given to individual-level and community-level SES among indigenous populations. This article contributes to the multilevel study of the social determinants of indigenous peoples’ health. Findings show that blood pressure significantly varied between communities in Greenland. Although small, the extent of geographic variation was similar to that observed in multilevel studies conducted among non-indigenous (and mostly urban) populations (16). It underlines the importance of considering the social determinants of health, across multiple levels, to understand variation in blood pressure among Greenlanders.

Among participants to the Greenland survey, education was not significantly associated with blood pressure. This absence of association could be due to low variability in the distribution of education, with an overrepresentation in less favourable categories. In this study, 60% of participants had a high school education or less. As indicated by Shepherd et al.'s review of SES and health studies among indigenous populations in Australia, observing social gradient in health is often contingent upon the SES measure used and the health outcome observed. Different measures of SES, such as personal income or occupational status, should be examined for their association with blood pressure and with cardiovascular risk factors and morbidity more generally.

As observed in studies conducted among non-indigenous populations (15–17), there was a direct association between community socio-economic conditions and SBP and DBP, independently of individuals’ characteristics. This speaks to the salience of community conditions as important determinants of cardiovascular health in indigenous populations (2). Specifically, there appears to be an inverse U-shaped association between socio-economic conditions at the community-level and elevated blood pressure, with lower SBP and DBP observed among participants living in communities with the lowest and highest disposable household income. This inverse U-shaped association represents transitions from more remote and traditional villages, but which are often more socioeconomically deprived, to more urbanized and affluent towns. It may reflect the protective role of traditional lifestyles with regard to country food consumption and social networks in remote villages, and better access to health-promoting resources, services and healthier social/cultural norms in wealthier towns. In the fully adjusted model, the remaining unexplained variation between communities in DBP suggests that other environmental conditions might be important to consider.

Measures of community conditions used in this study were limited by available data. More work is needed to better characterize the social, economic and built environment conditions of indigenous communities to understand the relevance of community context for cardiovascular health and risk factors, but also for other health outcomes. Observing association between health outcomes and community-level socio-economic conditions, but not with individual SES, perhaps reflects the role the community plays in nurturing health and wellness among indigenous populations, beyond individual idiosyncrasies.

Although the cross-sectional design prevents analysing processes of causation, findings nonetheless contribute novel evidence about the social determinants of CVD among indigenous populations. More research is needed to understand how socio-environmental determinants “get under the skin” to influence health (1,14). Daniel et al. suggested three possible mechanisms: behaviours, psychosocial factors and stress responses axes (1,14). As indicated in recent studies, it is possible that behavioural risk factors mediate the association between community conditions and blood pressure (39). For example, a more traditional lifestyle in remote villages might be associated with a healthy blood pressure. Psychosocial factors, such as mastery, control and social support, are likely to be influenced by wider community conditions and, in turn, influence cardiovascular health, perhaps differently across towns and villages in Greenland. Living in communities in the middle of social and cultural transition, that is, between more remote villages and affluent towns, may act as a chronic environmental stressor influencing cardiovascular health through physiological stress responses, such as elevated allostatic load. Future studies should examine the mediating role of behavioural, psychosocial and physiological stress responses in the association between socio-environmental conditions and cardiovascular health among indigenous populations.

There was a tendency for SES measured at the individual and community levels to be more strongly associated with blood pressure among men than in women. This possibly indicates differential gender effects, with men's blood pressure being more influenced by the conditions of their surrounding environment, expressed by lower blood pressure in more traditional or more urban, and affluent, communities. It is possible that women may have other health responses to being exposed to adverse socio-economic conditions. Future studies should examine gender-based health inequalities in relation to SES.

### Public health implications

Findings of this study show that blood pressure varies significantly between communities in Greenland and are consistent with studies among general populations where higher community-level SES is associated with better health. However, our findings also indicate the protective role of remote and more traditional settlements in relation to this risk factor for CVD in Greenland. As such living in communities at both end of the “spectrum” of settlement type and socio-economic conditions, that is, in remote traditional villages and affluent and more urbanized towns, seems to be associated with healthier blood pressure among Greenlanders.

In this large representative sample of Greenlanders, the unadjusted prevalence of hypertension is high (38%), suggesting that it is an important population health challenge in relation to CVD in this country. Ecological interventions targeting community conditions that shape the distribution cardiovascular risks and diseases are required to yield population-wide health benefits. Results of this study suggest that public health and social policies, programmes and interventions aiming to improve living conditions might improve cardiovascular health in Greenland. These interventions could be first directed to communities “in the middle” of social and cultural transition, where the population appears to be more at-risk for high blood pressure. These interventions should be developed alongside individual level health promotions efforts to encourage the pursuit of traditional lifestyles.
